# Bacterial and archaeal communities reflect long-term pH shift induced by ash fertilization in boreal peatland forest soils

**DOI:** 10.1093/femsec/fiag105

**Published:** 2026-09-09

**Authors:** Matilda Kattilakoski, Jenni Hultman, Krista Peltoniemi, Paavo Ojanen, Päivi Väänänen

**Affiliations:** Natural Resources Institute Finland (Luke), 00790 Helsinki, Finland; Department of Microbiology, University of Helsinki, 00790 Helsinki, Finland; Natural Resources Institute Finland (Luke), 00790 Helsinki, Finland; Natural Resources Institute Finland (Luke), 00790 Helsinki, Finland; Natural Resources Institute Finland (Luke), 00790 Helsinki, Finland; Department of Forest Sciences, University of Helsinki, 00790 Helsinki, Finland; Natural Resources Institute Finland (Luke), 00790 Helsinki, Finland

**Keywords:** drained peatland forest, archaea, bacteria, ash fertilization, microbial community, boreal forest soil

## Abstract

Ash fertilization is a forest management practice used to improve forest growth in boreal peatland forests. It impacts soil chemistry, vegetation, and soil organisms, yet little is known about its long-term impacts on soil microbial communities. Here, we focus on the long-term impacts of ash fertilization on the soil microbiome of drained boreal peatland forests in Finland. We hypothesized that the ash-induced changes in soil chemistry have led to long-term changes in soil microbial communities. To study this, we analyzed soil bacterial and archaeal communities from seven drained peatland forest sites in Finland. Each site had an ash fertilized plot (fertilized 8−43 years before sampling) and an unfertilized plot. Soil pH was higher at ash fertilized plots in five out of the seven sites. *Pseudomonadota, Bryobacteraceae*, and *Planctomycetaceae* were more abundant in samples from the control plots, whereas *Actinomycetota* were more abundant in samples from the ash fertilized plots. According to distance-based redundancy analysis, soil pH was important driver of soil bacterial and archaeal community structure. Site explained a greater proportion of community variation than treatment. Therefore, impacts of wood ash on soil in peatland forest stands should be considered by taking into account their site-specific factors and management history.

## Teaser

Long-term effects of ash fertilization on soil bacterial and archaeal communities in drained boreal peatland forests in Finland.

## Introduction

Drained peatland forests are economically and ecologically significant in Finland as they cover around one-fifth of forestry land and play a crucial role in carbon dynamics (Korhonen et al. 2024). Due to their high organic matter content and waterlogged conditions, peat soils act as carbon sinks when undrained but pose a risk of carbon loss from soil when drained (Turunen et al. 2002, Ojanen et al. 2013). With over half of Finnish peatlands drained for forestry, sustainable management practices that minimize carbon loss from soil are essential (Nieminen et al. 2018).

One such practice is wood ash fertilization (Lehtonen et al. 2021), a method increasingly applied in Finnish forestry to provide long-term boost (lasting up to 30 years following fertilization) to tree growth (Hökkä et al. 2024). Wood ash, rich in mineral nutrients like potassium (K) and phosphorus (P) but lacking nitrogen (N), is particularly effective in boosting tree growth in acidic peat soils where N is not the limiting factor. Lack of P and K typically limit tree growth in peatland forests with thick peat layer. Ash fertilization also shifts the ground vegetation towards more fertile vegetation types and increases litter production, which further influences soil chemistry (Ojanen et al. 2019, Hotanen 2025).

In addition to shifts in nutrients, alkaline ash quickly raises soil pH. In boreal peatland forests, including those in Finland, soil pH does not directly regulate tree growth; instead, growth responses are primarily controlled by hydrology–driven oxygen availability and nutrient supply, with pH influencing growth only indirectly through biogeochemical processes (Huotari et al. 2015, Lampela et al. 2023, Tong et al. 2024). However, pH is a major determinant of microbial community structure in soil (Fierer and Jackson 2006, Lauber et al. 2009, Kaiser et al. 2016), and therefore ash fertilization may substantially reshape microbial communities. The shifts in soil chemistry induced by ash fertilization influence a variety of organisms, including mosses, shrubs, lichens, and critically, soil microbiome (Huotari et al. 2015). As soil microbial communities play a key role in nutrient and greenhouse gas cycling, organic matter decomposition, and carbon storage, it is crucial to understand the changes in microbiome induced by ash fertilization.

Previous studies have reported that the effects of ash fertilization on soil microbial communities vary depending on site, ash dose, and ash form (Perkiömäki and Fritze 2002, Noyce et al. 2016, Peltoniemi et al. 2016, Bang-Andreasen et al. 2017, Smenderovac et al. 2022, Smith et al. 2024). In mineral soils, increase in microbial activity, bacterial numbers and growth rate of soil microorganisms has been reported (Fritze et al. 2000, Perkiömäki and Fritze 2002, Zimmermann and Frey 2002, Bang-Andreasen et al. 2017) with copiotrophic groups increasing and oligotrophic and acidophilic groups decreasing after ash fertilization (Bang-Andreasen et al. 2017, 2020, 2021). In oligotrophic peatland forests, decrease in microbial biomass has been reported after ash fertilization (Björk et al. 2010). Some studies have reported only minimal changes in the microbial community composition (Björk et al. 2010, Noyce et al. 2016, Smenderovac et al. 2022). Impacts of ash fertilization on microbial communities have mostly been studied in mineral soils and in short-term (≤ 8 years after fertilization). However, ash fertilization is known to impact tree growth for decades after the application. There is a lack of long-term data on the effects on soil microbial communities in drained peatland forest soils, especially using state-of-the-art DNA-based techniques (Huotari et al. 2015).

Here, we explore long-term (8–43 years after fertilization) impacts of wood ash fertilization on the soil bacterial and archaeal communities of drained boreal peatland forests in Finland to better understand the effects of this forest management action. We hypothesize that ash-induced changes in soil pH and nutrient status lead to lasting shifts in bacterial and archaeal community compositions.

## Materials and methods

### Site description and sampling

**Figure 1:**
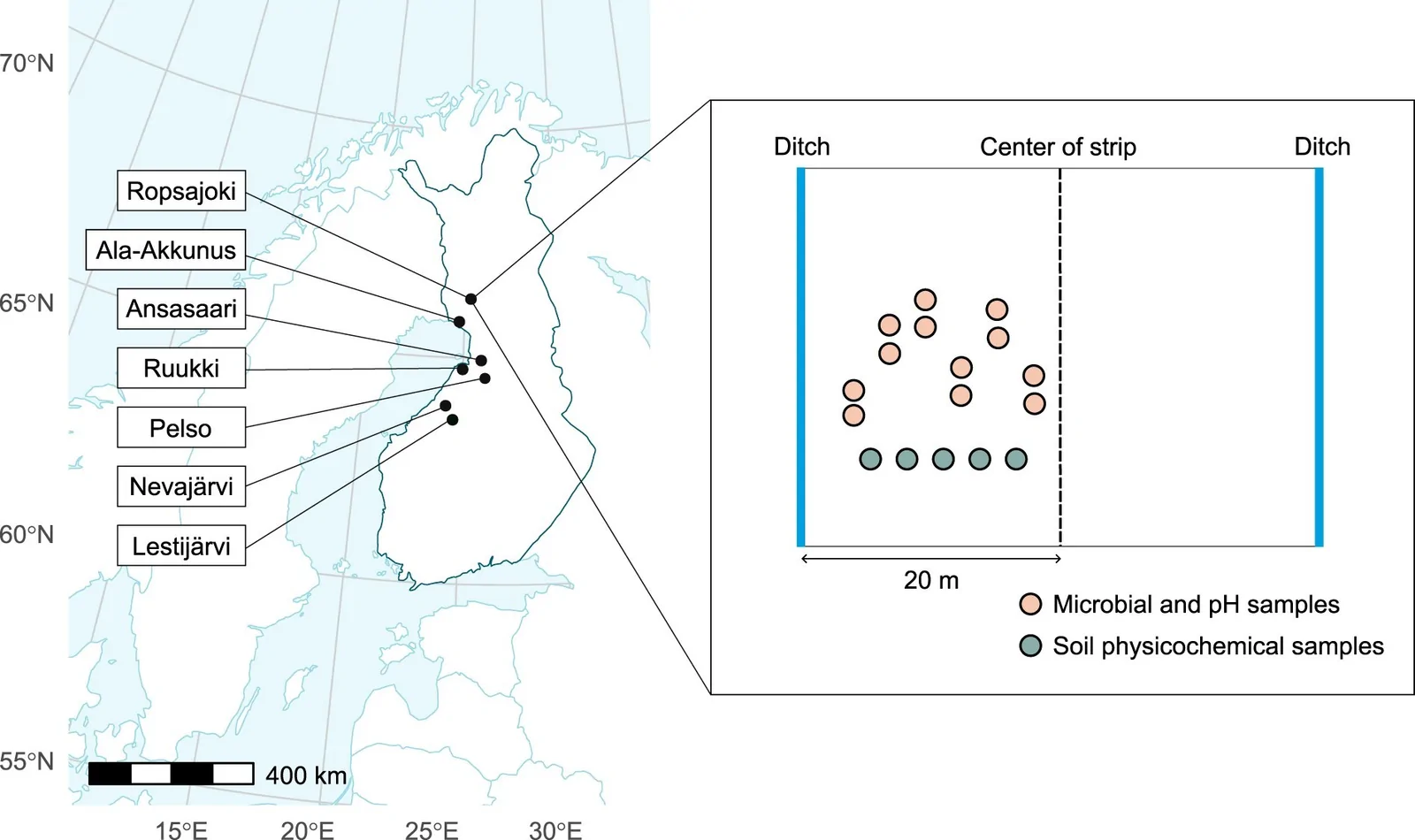
Locations of the sampling sites in Finland and a sampling scheme showing the approximate locations of the soil sampling points within a treatment plot.

The study consisted of seven peatland forest sites located in central and northern Finland where long-term ash fertilization experiments had been carried out (Fig. 1). The sites represented three drained peatland forest types with varying nutrient levels (Table 1) classified following Laine et al. (2012). These forest types are from most to least fertile *Vaccinium myrtillus, Vaccinium vitis*-*idaea*, and dwarf shrub peatland forest. Detailed description of vegetation can be found in Hotanen (2025). Ash fertilization shifted the site towards a more fertile forest type at Ala-Akkunus and Ruukki. All sites had a peat layer thicker than 30 cm and had been fertilized 8–43 years prior to sampling. The applied ash amounts were similar to what is applied in practical forestry (Table 1).

**Table 1:** Description of the sampling sites and treatments. Mtkg = *Vaccinium myrtillus* drained peatland forest type, Ptkg = *Vaccinium vitis-idaea* drained peatland forest type, and Vatkg = dwarf shrub drained peatland forest type.

Site	Peatland forest type (control)	Peatland forest type (fertilized)	Fertilization year(s)	Ash amount (kg ha⁻¹)	Drainage year(s)	Ash type	P amount (kg ha⁻¹)	K amount (kg ha⁻¹)	Ca amount (kg ha⁻¹)
Ropsajoki	Mtkg	Mtkg	1980	5000	1961	Granulated wood ash	135	224	1414
Ala-Akkunus	Ptkg	Mtkg	1978, 2002	5000, 5000	1977	Loose wood ash	43	171	1386
Ansasaari	Ptkg	Ptkg	2014	5000	1969, 2007	Granulated wood ash	45	117	698
Ruukki	Vatkg	Mtkg	1985	5000	1929, 1981	Loose wood ash	135	225	1440
Pelso	Vatkg	Vatkg	1997	5000	1930, 1994	Granulated wood ash	25	85	465
Nevajärvi	Ptkg	Ptkg	2014	5000	1926, 2006	Granulated wood ash	45	117	698
Lestijärvi	Vatkg	Vatkg	1979	10 000	1977	Loose bark ash	15	42	441

All sites had been previously drained for forestry by ditching. Each site has an ash fertilized plot and an adjacent unfertilized control plot. Ash had been applied once to all sites except to Ala-Akkunus that was fertilized twice due to the low quality of ash used the first time.

Lestijärvi had received twice the amount of ash compared to the other sites, to compensate for the low nutrient levels of the used ash. Regardless of this, the nutrient amounts were lower in Lestijärvi compared to the other sites. Each plot consisted of 12 sampling spots located at varying distances (4.2–22.9 m) from the drainage ditch (Fig. 1). This distance influences soil moisture, as the water table level tends to be lower closer to the ditch.

For microbiological analyses, a total of 168 soil samples were collected from the top 10 cm of soil, which typically contains the highest microbial biomass, activity, and diversity, as well as the strongest soil nutrient responses to ash fertilization. Samples were collected below loose litter and green living vegetation using a soil auger (63 mm × 41 mm). Soil samples were placed in individual bags and homogenized by hands before freezing. The samples were collected at the end of the growing season in September 2021 (Ruukki and Pelso) and in September 2022 (rest of the sites), 8–43 years after the ash fertilization. All the sampling equipment were sterilized between every sample using 70% ethanol. Samples were put on ice on site, kept cool and stored frozen at −20°C within 48 h until DNA extraction.

### Soil physicochemical analysis

For pH measurements, soil samples were collected from the top 10 cm at same locations as microbiological samples. The pH was measured in water suspension (soil:water 15:25 v/v). Separately from the microbiological samples, soil samples for other soil physicochemical analyses were collected at each plot from the top 10 cm at even distances in five locations from the center of the strip towards the ditch (Fig. 1, Tables S1 and S2). Concentrations of nutrients were measured using inductively coupled plasma optical emission spectroscopy (iCAP 6000 series, Thermo Fisher). Soil moisture was measured with LECO TGA-701 and soil carbon and nitrogen content with LecoCN TruMac®-analyzer (Leco Corporation, USA). Soil organic matter content was measured as loss on ignition.

### Microbial DNA extraction and sequencing

From each soil sample, one 250 mg representative subsample was collected for DNA extraction. Whole community DNA was extracted from the soil samples (*n* = 168) using DNeasy PowerSoil Pro Kit (QIAGEN, Germany) and following the associated protocol (QIAGEN 2021). Sample homogenization was done with TissueLyser III (QIAGEN). DNA concentrations were measured using the Qubit^TM^ Flex Fluorometer (Invitrogen). DNA samples were stored at −20°C until sequencing. Metagenomic libraries were constructed using Nextera XT DNA Library Preparation Kit (Illumina, USA) and sequenced from all samples using Illumina NovaSeq 6000 with S4 flow cell by Novogene (Cambridge, UK).

### Read-based taxonomic profiling

The raw sequence data was filtered by Novogene by removing sequencing adapters, reads containing >10% undetermined bases and reads containing >50% low quality bases (Q score ≤ 5). The quality of the sequence reads was assessed using fastQC v0.11.9 (Andrews 2010) and MultiQC v1.19 (Ewels et al. 2016). Following a read-based approach, the sequence reads were assigned taxonomic annotation using phyloFlash v3.4.2 (Gruber-Vodicka et al. 2020) with SILVA database release 138.2 (Quast et al. 2013).

### Data analysis and statistics

Data analysis was done using R v. 4.4.2 (R Core Team 2024) focusing on archaeal and bacterial reads. Differences in community structure between samples were visualized using principal coordinate analysis (PCoA) based on Bray–Curtis distances calculated from relative abundances of phyloFlash Nearest Taxonomic Units, representing the lowest assignable taxonomic rank. Pairwise differences in community composition between treatments, between peatland forest types, and between sites were tested using permutational multivariate analysis of variance (PERMANOVA) (function *adonis2*, R-package vegan v2.6–10; Oksanen et al. 2001) on a Bray–Curtis dissimilarity matrix. Permutations (*n* = 999) were restricted within blocks defined by the interaction of site and treatment, the interaction of peatland forest type, site and treatment or by treatment depending on the comparison, to account for the experimental design. Differences in community composition between sites among all samples were tested using PERMANOVA (function *adonis2*) on a Bray–Curtis dissimilarity matrix with permutations (*n* = 999) restricted within blocks defined by treatment. The *P*-values were adjusted using Benjamini–Hochberg method. Differences in community composition between treatments within each site were statistically tested using pairwise PERMANOVA (function *pairwise.adonis*, R-package pairwiseAdonis; Martinez Arbizu 2020).

Differences in the relative abundances of reads assigned to individual bacterial and archaeal families and phyla (later referred to as relative abundance) between treatments were tested using linear mixed-effects model (function *lme*, R-package nlme v3.1.166; Pinheiro et al. 2025), with treatment as a fixed effect and random intercepts for site and treatment nested within site. The *P*-values were adjusted using Benjamini–Hochberg method. The relationship between community structure and selected environmental variables (pH and distance from ditch), which were measured at all microbiological sampling locations (Kattilakoski et al. 2025), was analysed using distance-based redundancy analysis (dbRDA; function *dbrda*, R-package vegan v2.6–10; Oksanen et al. 2001). Other environmental variables were not included because they were measured at different sampling locations than the microbiological samples.

Differences in soil characteristics between treatments within sites were tested using the Mann–Whitney U test. The *P*-values were adjusted using Benjamini–Hochberg method. Differences in soil characteristics between control plots among sites were tested using one-way ANOVA. Then, linear mixed–effects models were used to estimate temporal changes in ash fertilization effects on soil physicochemical variables. Fixed effects included treatment (ash fertilized vs. control; describing the initial impact of fertilization) and an interaction between ash fertilization and years since fertilization, describing the temporal development of the fertilization impact. Site and treatment nested within site were included as random intercepts. Variance components were estimated using an intercept-only linear mixed-effects model, with site and treatment nested within site as random effects.

## Results

### Soil physicochemical characteristics

**Figure 2:**
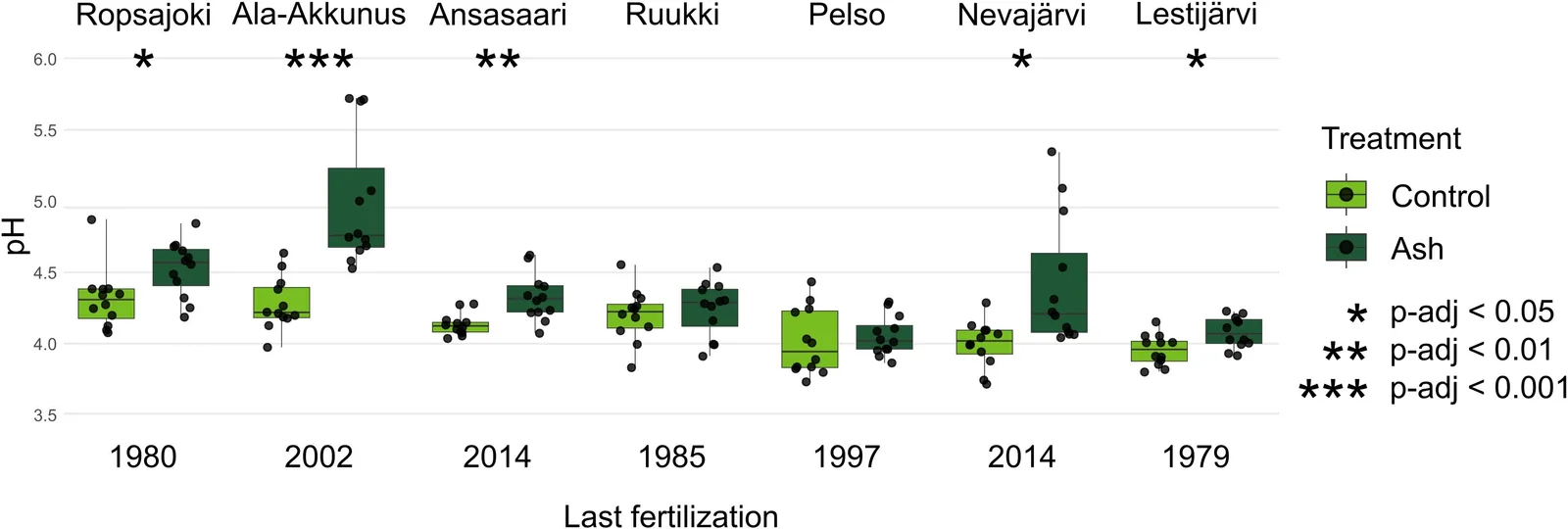
Soil pH in the 0−10 cm soil layer at the ash fertilized and control plots across sites (Mann–Whitney U test). Asterisks (*) indicate statistically significant difference between control and ash fertilized plots.

Soil pH ranged from acidic (3.71) to mildly acidic (5.72) and was significantly higher (*P* < .05, *P*-adj < .05) in samples from ash fertilized plots compared to control plots at all sites except Pelso and Ruukki (Fig. 2). Mean soil pH differed significantly among control sites (one-way ANOVA, F_6,77_ = 8.07, *P* < .001).

Soil carbon content varied between 42.3% and 55.9% while soil nitrogen content ranged from 1.01% to 2.91% (Table S1). In Nevajärvi, soil carbon content was lower (*P* < .05, *P*-adj = .15) in samples from the ash fertilized plot compared to the control plot. No other site showed a significant difference in soil carbon content between treatments. Mean soil carbon content differed significantly among control sites (one-way ANOVA, F_6,28_ = 10.52, *P* < .001). None of the sites showed significant differences in soil nitrogen content between treatments (Table S1). Mean soil nitrogen content differed significantly among control sites (one-way ANOVA, F_6,28_ = 11.35, *P* < .001).

Soil organic matter content varied between 75.7% and 98.8%. In Ansasaari, Nevajärvi and Ala-Akkunus it was slightly lower (*P* < .05, *P*-adj = .06 for Nevajärvi and Ala-Akkunus, *P*-adj = .07 for Ansasaari) in samples from the ash fertilized plot compared to control plot. Mean soil organic matter content differed significantly among control sites (one-way ANOVA, F_6,28_ = 7.19, *P* < .001).

Soil potassium concentration varied between 268 mg kg^−1^ and 4760 mg kg^−1^ and was higher in samples from ash fertilized plots compared to control plots in Ala-Akkunus (*P* < .05, *P*-adj < .05), Ansasaari (*P* < .05, *P*-adj < .05) and Ropsajoki (*P* < .05, *P*-adj < .05). Soil phosphorus concentration varied between 493 mg kg^−1^ and 4100 mg kg^−1^ and was higher in samples from ash fertilized plots compared to control plots in Ala-Akkunus (*P* < .01, *P*-adj = .06) and Ropsajoki (*P* < .05, *P*-adj = .11). Both mean soil potassium (one-way ANOVA, F_6,28_ = 2.96, *P* < .05) and mean soil phosphorus (one-way ANOVA, F_6,28_ = 11.51, *P* < .001) concentrations differed significantly among control sites.

Temporal changes in fertilization effects varied among soil variables (Table S3). Ash fertilization effects increased significantly over time for soil carbon content and soil organic matter content, although the magnitude of these changes was small (estimates 0.093 and 0.12, respectively). No significant temporal trends were detected for other soil physicochemical variables.

Across soil physicochemical parameters, the majority of estimated variability occurred at the sample level. Site-level variability was strongest for soil nitrogen content and soil moisture while treatment-level variability was strong for pH, soil carbon content, and soil organic matter content (Table S4). Treatment plot–level variability exceeded site–level variability for soil carbon content, soil organic matter, soil phosphorus and potassium concentrations, and soil pH, indicating a meaningful effect of the treatment on these variables.

### Bacterial and archaeal community composition

**Figure 3:**
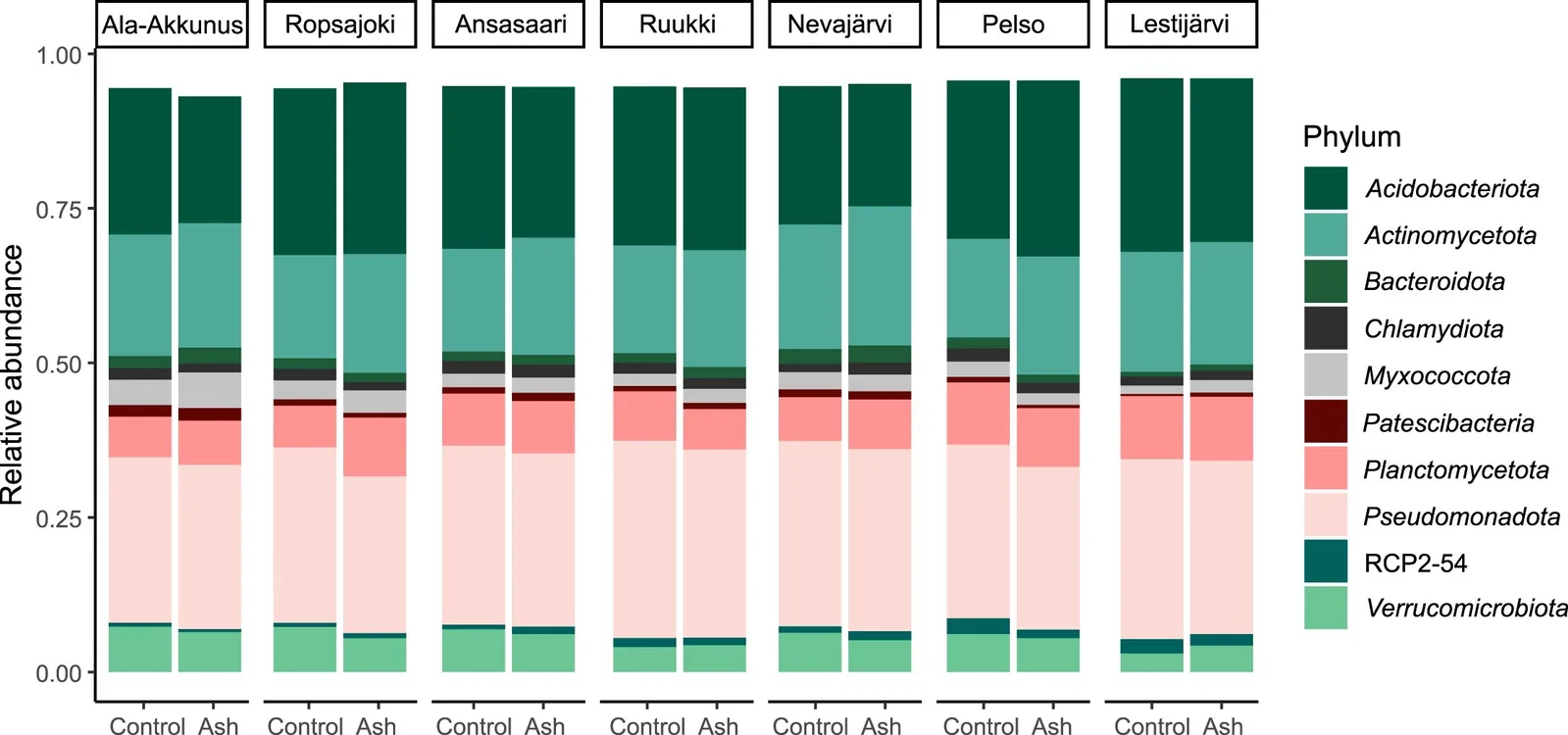
Relative abundances of the 10 most abundant bacterial phyla across peatland forest sites and treatments.

The community composition of the ten most abundant bacterial and archaeal phyla was similar between treatments and sites (Fig. 3). The compositions were similar on order and family level (Figs S1 and S2). *Pseudomonadota, Acidobacteriota*, and *Actinomycetota* were the most abundant phyla. There were only bacterial taxa within the ten most abundant phyla, orders, and families.

**Figure 4:**
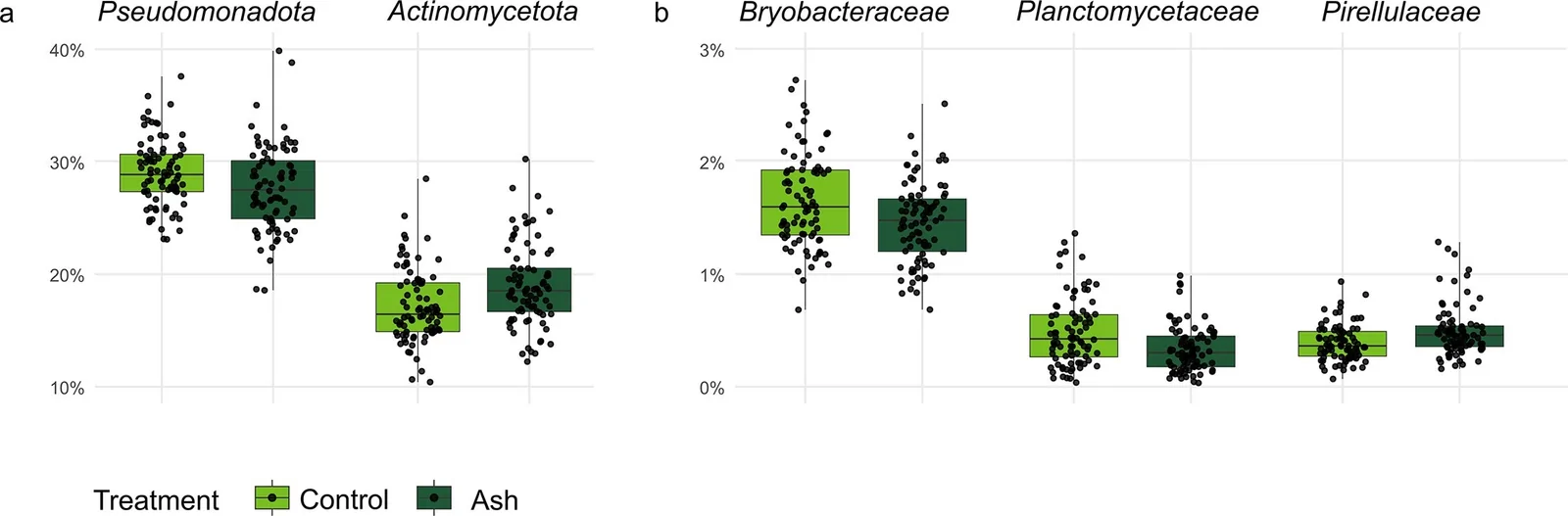
(a) Abundances of the abundant bacterial phyla (mean abundance twice the mean of all phyla or greater) with differences between treatments. (b) Abundances of the abundant bacterial families (mean abundance twice the mean of all families or greater) with differences between treatments.

When comparing the relative abundances of abundant phyla (mean abundance twice the mean of all phyla or greater) between treatments and across all samples, we found that *Pseudomonadota* were marginally (*P* < .05, *P*-adj = .07) more abundant in samples from control than from ash fertilized plots. *Actinomycetota* were slightly (*P* < .05, *P*-adj = .06) more abundant in samples from ash fertilized plots compared to samples from control plots (Fig. 4a). No significant differences between treatments were observed in the abundances of abundant archaeal phyla.

There were minor differences in the relative abundances of abundant bacterial families (mean abundance greater than two-fold mean abundance of all families) between treatments (Fig. 4b). *Bryobacteraceae* and *Planctomycetaceae* were marginally (*P* < .01 and *P* < .05, respectively) less abundant in ash fertilized plots. In contrast, *Pirellulaceae* were slightly (*P* = .06) more abundant in ash fertilized plots. None of these differences remained significant after *P*-value adjustment. No significant differences between treatments were observed in the abundances of abundant archaeal families.

**Figure 5:**
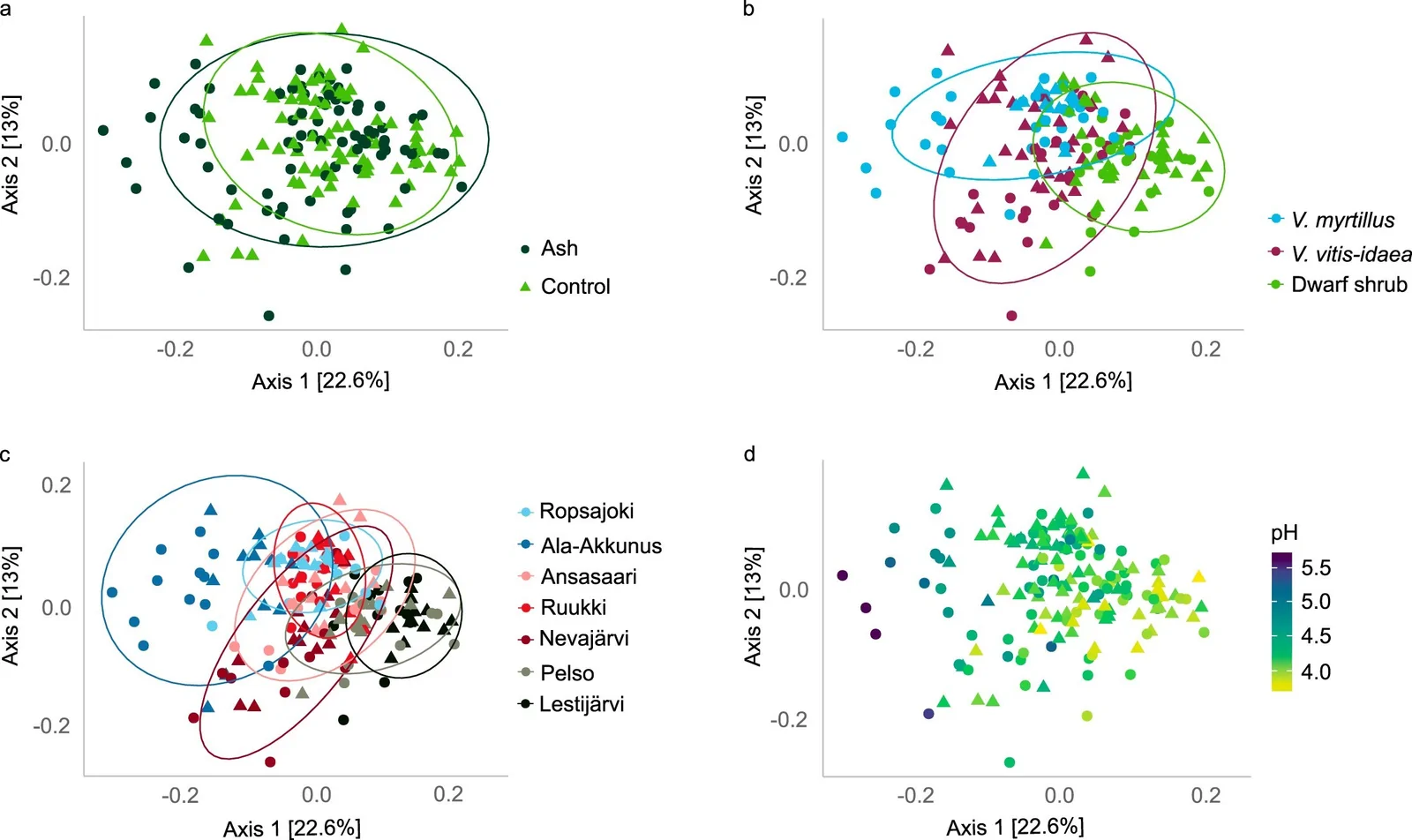
PCoA of sample level soil archaeal and bacterial community composition (a) between treatments, (b) between different peatland forest types, (c) between different sites, and (d) across a soil pH gradient. The ellipses represent 95% confidence intervals.

Treatment (control vs. fertilization) did not explain any of the variation in bacterial and archaeal communities within all samples according to PERMANOVA (Fig. 5a). Based on PCoA, bacterial and archaeal communities in different drained peatland forest types appeared to differ from each other (Fig. 5b). However, PERMANOVA showed that none of the peatland forest type pairs significantly differed from each other based on their community composition. Bacterial and archaeal communities differed between peatland forest sites according to PERMANOVA (*P* < .01). While not separating from each other distinctly in a PCoA (Fig. 5c), PERMANOVA estimated that site explained 11%–39% of the variation in bacterial and archaeal community composition between two sites. Treatment explained 0%–12% of the variation within the same site pairs, always explaining less variation than site (Table S5). Among all samples, site explained 9% of the variation in the community composition according to PERMANOVA (*P* < .01). There was also a clear gradient in pH along the horizontal axis (Fig. 5d).

**Figure 6:**
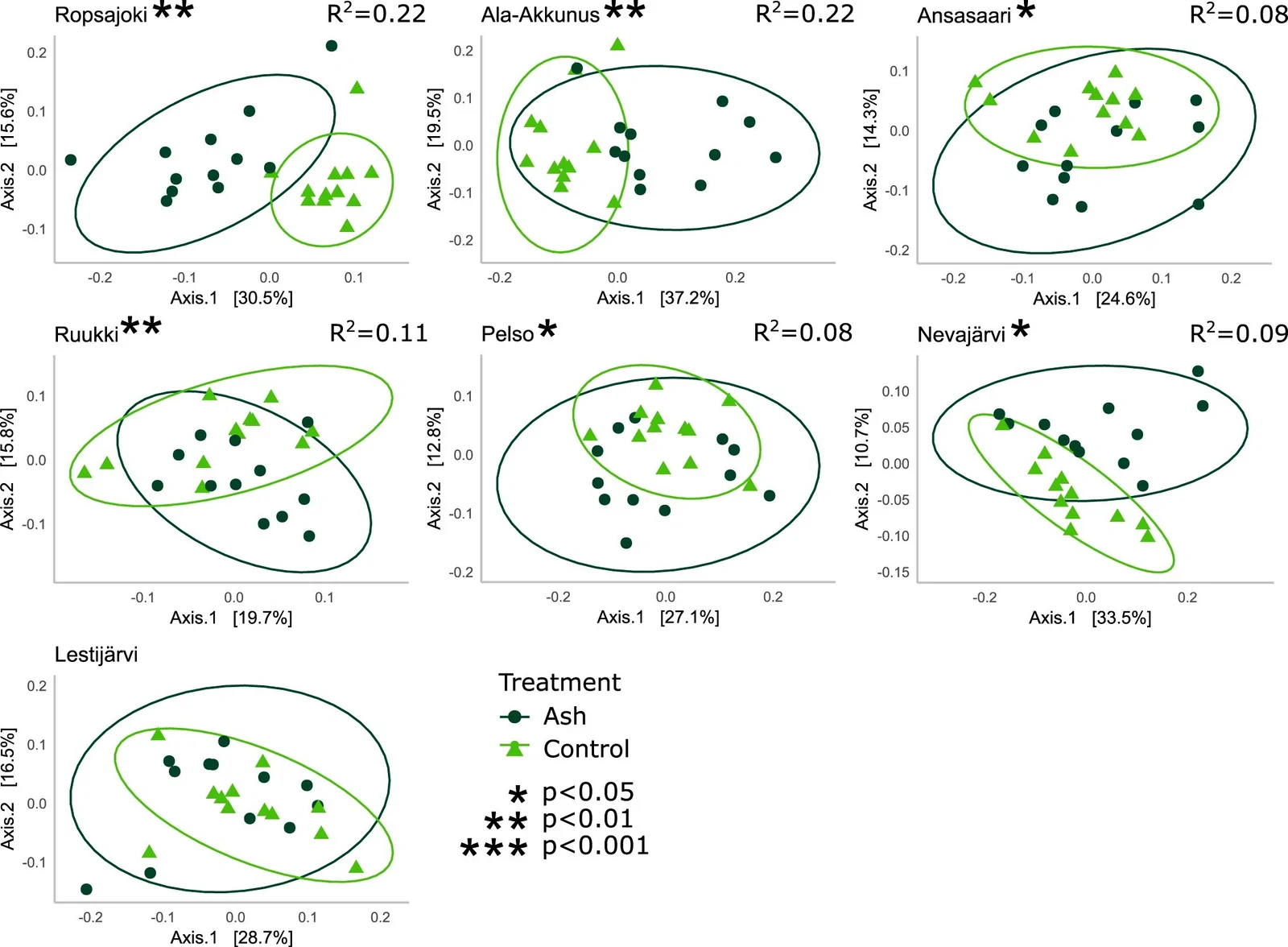
PCoA of soil archaeal and bacterial community composition between treatments within each site (pairwise PERMANOVA). The ellipses represent 95% confidence intervals.

Within individual sites, bacterial and archaeal communities differed significantly between ash fertilized and control plots, with treatment explaining 8%–22% of the variation, except in Lestijärvi (*P* = .1; Fig. 6).

### The relationship between community structure and soil pH

**Figure 7:**
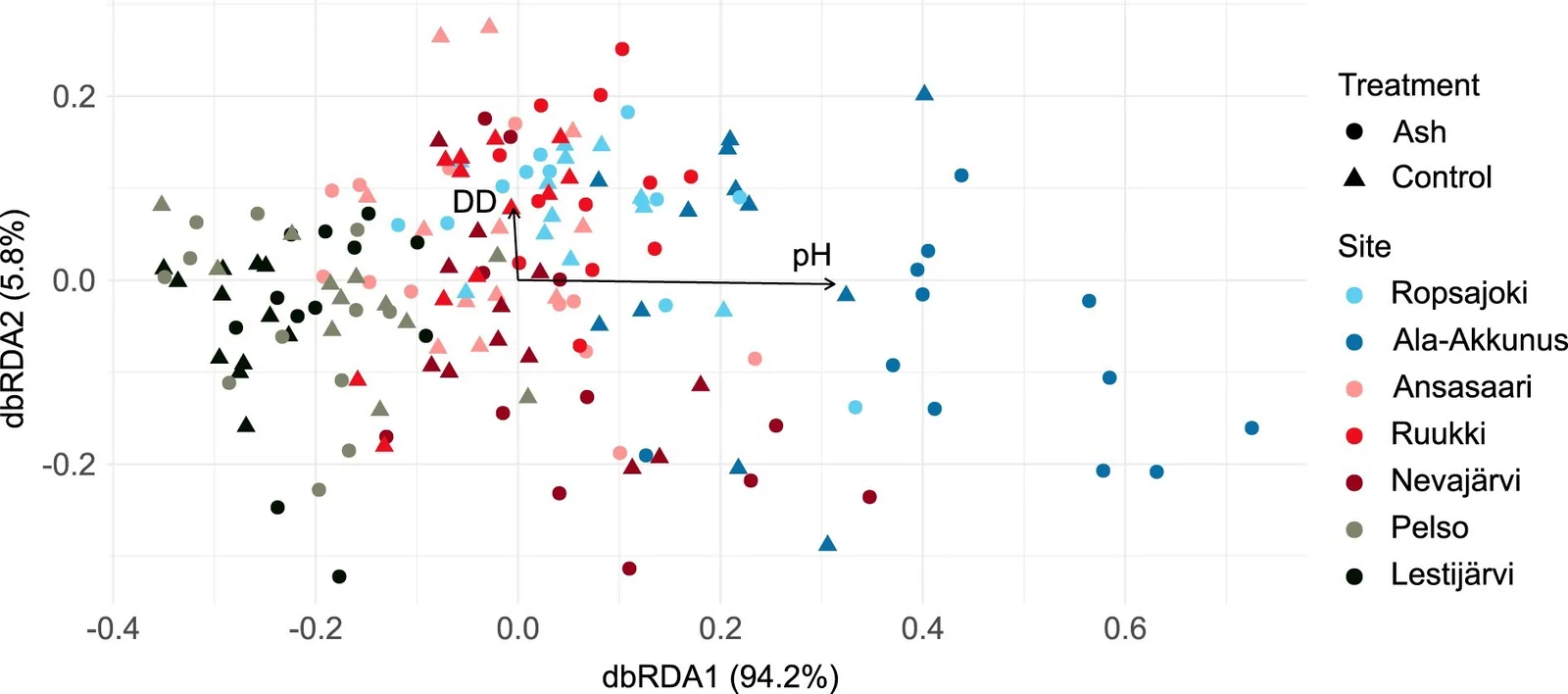
dbRDA showing the relationship between bacterial and archaeal community composition and the environmental variables included in the model [soil pH and distance from ditch (DD)].

The dbRDA model including soil pH and distance from ditch explained 9% of the variation in community composition (adjusted *R*^2^ = 0.09, F = 9.58, *P* < .01, Fig. 7). Of the two variables, only soil pH significantly explained the variation (F = 18.00, *P* < .01) whereas distance from ditch was not significant (F = 1.16, *P* = .25).

## Discussion

Our results show that soil pH remained higher at five of the seven fertilized sites compared to their respective control sites, even 8–43 years after ash application. This is consistent with previous research on the long-term effects of ash fertilization (Huotari et al. 2015). Given the widely recognized role of soil pH as one of the main drivers of soil bacterial community structure (Fierer and Jackson 2006, Lauber et al. 2009, Kaiser et al. 2016, Lladó et al. 2018), we expected to see a shift in the bacterial communities in ash fertilized sites.

Overall, however, the relative abundances of the ten most abundant bacterial phyla were similar across treatments and sites. *Pseudomonadota, Acidobacteriota*, and *Actinomycetota* were the most abundant phyla as commonly reported for soils globally (Janssen 2006, Delgado-Baquerizo et al. 2018). *Pseudomonadota* were more abundant in control samples with lower pH compared to samples from ash fertilized plots with higher pH. *Pseudomonadota* has been reported to have varying responses to increasing soil pH by different taxa within the phylum (Lauber et al. 2009, Bang-Andreasen et al. 2017, Lladó et al. 2018), suggesting that taxa present in our samples are likely adapted to acidic conditions. *Actinomycetota* on the other hand followed the trend of increasing abundance with increasing pH reported for the phylum (Lauber et al. 2009, Peltoniemi et al. 2016, Lladó et al. 2018).

In addition to pH, nutrient inputs, particularly phosphorus, may have contributed to the observed shifts. Richy et al. (2024) reported that phosphorus application together with nitrogen increased *Actinomycetota* and decreased *Pseudomonadota*, which aligns with our results. However, the overall differences in the abundances between treatments in our study were not drastic.

PERMANOVA analyses showed that treatment did not explain variation in archaeal and bacterial community composition when all samples were analysed together. Site explained small but statistically significant proportion of variation (9%), indicating that inter-site variability exceeded treatment-driven effects. Accordingly, ash-induced changes in community composition became more visible when sites were analysed individually, except in Lestijärvi, where the low nutrient content and type of the applied ash may have limited treatment effects. Thus, the high inter-site variability likely explains the lack of family-level differences when all samples were analyzed together.

The strongest treatment-level responses were observed at Ala-Akkunus, where the most recent application of loose ash had been carried out. In addition, this site was fertilized twice, because the nutrient content of the first application was low. This repeated fertilization of loose ash may have contributed to the greater plot-level differences in both soil physicochemical properties and microbial communities observed at this site.

In contrast, although soil pH was significantly elevated at Ansasaari and Nevajärvi, fertilization at these sites was relatively recent and granulated wood ash was applied. Granulated ash releases nutrients more slowly than loose ash, delaying its effects on soil chemistry and biota (Hytönen and Hökkä 2020). The ash type and fertilization time might explain the weaker responses in the soil properties and microbial communities at these sites.

Microbial communities in peatlands are influenced by physicochemical and hydrological characteristics, as well as vegetation. Because these factors interact and affect one another, it is difficult to isolate the impact of any single factor on microbial communities (Andersen et al. 2013). Moreover, these characteristics vary not only between peatland forest stands but also within individual stands. Differences among sites may originate from pre-drainage variation in peat properties and hydrological conditions. In addition, drainage and the following increase in tree growth alter peat nutrient availability, C:N ratio and bulk density (Laiho et al. 1999). The time since drainage further contributes to differences between sites as the drainage-induced succession in vegetation and soil proceeds.

At two sites, the peatland forest type had shifted due to fertilization, with more nutrient-rich forest types determined at ash fertilized plots. Vegetation shifts are known to influence soil microbial communities and may have contributed to the differences observed between plots at these sites (Le Geay et al. 2024). However, the vegetation shifts were observed between closely related peatland forest types and therefore represent relatively minor changes in vegetation structure or function. Consistent with this, microbial community compositions were relatively similar between different peatland forest types.

Due to the lack of pre-treatment data, our analyses rely on plot-level comparisons at the time of sampling. As a result, we cannot exclude the possibility that ash fertilized and control plots differed before any treatment was applied. However, treatment plots were located within the same peatland forest stand and shared a similar management history, with ash fertilization being the only management difference.

Overall, ash fertilization resulted in long-term increases in soil pH, that were significantly associated with bacterial and archaeal community composition. Spatial heterogeneity in site characteristics as well as differences in fertilization time, ash type, ash dose, and nutrient content likely explain the varying responses of soil bacterial and archaeal communities to ash fertilization observed among sites. Bacterial and archaeal community compositions did not distinctly differ between control and fertilized treatments but rather followed a gradient in pH. Community compositions varied more between the seven studied peatland forest sites than between control and ash fertilized plots or the three different peatland forest types. Therefore, impacts of wood-ash on soil in peatland forest stands should be considered by taking into account their site-specific factors and management history. Our results suggest that ash doses commonly applied in forest management do not lead to strong or persistent changes in soil bacterial and archaeal communities. Instead, substantial community overlap between treatments suggests that bacterial and archaeal communities stay relatively stable. Because peatland forests are important in long–term carbon storage and greenhouse gas regulation, further research is needed to understand how ash fertilization influences microbial functions in order to assess the broader climate impacts of this management practice.

## Supplementary Material

fiag105_Supplemental_File

## Data Availability

The sequences were deposited into European Nucleotide Archive (ENA) under the accession number PRJEB102797. Environmental data is available on Zenodo: https://doi.org/10.5281/zenodo.17727949.
