# Supplementary material for: Bacterial and archaeal communities reflect long-term pH shift induced by ash fertilization in boreal peatland forest soils
Source: FEMS Microbiol Ecol. 2026 Sep 9;102(10):fiag105. doi: 10.1093/femsec/fiag105 (PMC13592162; doi:10.1093/femsec/fiag105)
Supplement: Supplementary file 1 [file fiag105_supplemental_file.docx]

# **Bacterial and archaeal communities reflect long-term pH shift induced by ash fertilization in boreal peatland forest soils**

Matilda Kattilakoski^1,2,*^, Jenni Hultman^1^, Krista Peltoniemi^1^, Paavo Ojanen^1,3^, Päivi Väänänen^1^

^1^ Natural Resources Institute Finland (Luke), Helsinki, Finland

^2^ Department of Microbiology, University of Helsinki, Helsinki, Finland

^3^ Department of Forest Sciences, University of Helsinki, Helsinki, Finland

*Corresponding author: Department of Microbiology, University of Helsinki, Viikinkaari 9, 00790, Helsinki, Finland, [matilda.kattilakoski@helsinki.fi](mailto:matilda.kattilakoski@helsinki.fi)

**Supplementary tables S1–S5
Supplementary figures S1–S2**

Table S1. Metadata on soil physicochemical characteristics. SOM = Soil organic matter.

| **Site** | **Treatment** | **C (%)** | **N (%)** | **Soil moisture (%)** | **SOM (%)** | **K mg kg^-1^** | **P mg kg^-1^** |
| --- | --- | --- | --- | --- | --- | --- | --- |
| Ala-Akkunus | Control | 52.1 | 1.61 | 6.68 | 95.81 | 856 | 831 |
| Ala-Akkunus | Control | 53.2 | 1.45 | 6.66 | 96.17 | 725 | 756 |
| Ala-Akkunus | Control | 52.0 | 1.57 | 6.93 | 96.05 | 600 | 753 |
| Ala-Akkunus | Control | 51.6 | 1.53 | 6.93 | 95.73 | 685 | 785 |
| Ala-Akkunus | Control | 51.3 | 1.91 | 7.26 | 95.16 | 439 | 863 |
| Ala-Akkunus | Ash | 49.7 | 1.79 | 7.52 | 90.33 | 1050 | 1120 |
| Ala-Akkunus | Ash | 49.8 | 1.76 | 7.99 | 91.83 | 1100 | 1310 |
| Ala-Akkunus | Ash | 49.6 | 1.60 | 7.66 | 92.06 | 1050 | 1250 |
| Ala-Akkunus | Ash | 51.7 | 1.80 | 7.40 | 92.74 | 1270 | 1230 |
| Ala-Akkunus | Ash | 52.5 | 1.73 | 7.61 | 95.32 | 819 | 966 |
| Ansasaari | Control | 53.5 | 1.58 | 7.02 | 95.40 | 271 | 589 |
| Ansasaari | Control | 51.8 | 1.47 | 7.33 | 95.41 | 473 | 772 |
| Ansasaari | Control | 51.6 | 1.42 | 7.51 | 96.27 | 320 | 832 |
| Ansasaari | Control | 54.3 | 1.65 | 7.17 | 96.99 | 437 | 596 |
| Ansasaari | Control | 54.9 | 1.36 | 6.76 | 98.24 | 609 | 580 |
| Ansasaari | Ash | 50.8 | 1.33 | 7.41 | 92.45 | 739 | 794 |
| Ansasaari | Ash | 42.5 | 1.34 | 6.92 | 78.51 | 2210 | 2380 |
| Ansasaari | Ash | 52.7 | 1.17 | 6.76 | 95.90 | 580 | 562 |
| Ansasaari | Ash | 49.1 | 1.58 | 6.73 | 85.38 | 1390 | 1490 |
| Ansasaari | Ash | 52.1 | 1.53 | 7.11 | 94.09 | 809 | 963 |
| Nevajärvi | Control | 54.9 | 1.64 | 8.53 | 98.58 | 600 | 800 |
| Nevajärvi | Control | 54.9 | 1.73 | 7.37 | 97.74 | 599 | 881 |
| Nevajärvi | Control | 54.6 | 1.74 | 7.30 | 96.54 | 821 | 916 |
| Nevajärvi | Control | 53.2 | 1.44 | 7.39 | 98.01 | 528 | 716 |
| Nevajärvi | Control | 55.3 | 1.53 | 7.01 | 97.86 | 595 | 682 |
| Nevajärvi | Ash | 51.7 | 1.33 | 7.24 | 97.66 | 732 | 750 |
| Nevajärvi | Ash | 51.6 | 1.55 | 6.83 | 92.41 | 81 | 1390 |
| Nevajärvi | Ash | 51.8 | 1.40 | 7.08 | 94.72 | 758 | 883 |
| Nevajärvi | Ash | 42.3 | 1.22 | 6.03 | 75.69 | 3080 | 2590 |
| Nevajärvi | Ash | 53.4 | 1.61 | 6.73 | 96.03 | 649 | 951 |
| Ropsajoki | Control | 50.6 | 1.69 | 6.71 | 96.30 | 461 | 921 |
| Ropsajoki | Control | 50.9 | 2.03 | 6.43 | 95.02 | 524 | 1030 |
| Ropsajoki | Control | 51.6 | 1.86 | 6.76 | 95.83 | 528 | 974 |
| Ropsajoki | Control | 50.5 | 2.11 | 6.82 | 93.97 | 444 | 1070 |
| Ropsajoki | Control | 49.8 | 2.21 | 6.73 | 91.45 | 604 | 1140 |
| Ropsajoki | Ash | 50.8 | 1.85 | 6.56 | 94.42 | 557 | 1090 |
| Ropsajoki | Ash | 48.9 | 2.08 | 6.89 | 92.19 | 693 | 1530 |
| Ropsajoki | Ash | 50.3 | 1.93 | 6.56 | 94.06 | 776 | 1360 |
| Ropsajoki | Ash | 52.0 | 1.70 | 6.02 | 95.76 | 744 | 1080 |
| Ropsajoki | Ash | 51.0 | 2.16 | 6.19 | 92.99 | 886 | 1520 |
| Pelso | Control | 52.9 | 1.13 | 8.49 | 98.31 | 527 | 555 |
| Pelso | Control | 53.7 | 1.20 | 8.30 | 97.79 | 1040 | 639 |
| Pelso | Control | 53.7 | 1.19 | 8.87 | 97.49 | 653 | 678 |
| Pelso | Control | 52.7 | 1.33 | 8.89 | 97.03 | 520 | 625 |
| Pelso | Control | 52.3 | 1.01 | 9.51 | 98.75 | 578 | 572 |
| Pelso | Ash | 52.0 | 1.61 | 8.29 | 96.18 | 434 | 876 |
| Pelso | Ash | 52.6 | 1.09 | 9.13 | 98.07 | 614 | 570 |
| Pelso | Ash | 52.6 | 1.36 | 8.50 | 96.23 | 650 | 724 |
| Pelso | Ash | 54.6 | 1.86 | 7.55 | 96.77 | 412 | 850 |
| Pelso | Ash | 50.1 | 1.17 | 8.71 | 91.59 | 1220 | 1010 |
| Ruukki | Control | 53.0 | 1.92 | 8.36 | 92.62 | 404 | 959 |
| Ruukki | Control | 53.1 | 2.04 | 8.48 | 94.34 | 599 | 956 |
| Ruukki | Control | 51.4 | 1.85 | 8.78 | 94.23 | 425 | 910 |
| Ruukki | Control | 53.4 | 1.87 | 8.61 | 96.71 | 550 | 803 |
| Ruukki | Control | 51.3 | 2.91 | 8.16 | 90.70 | 268 | 1410 |
| Ruukki | Ash | 51.1 | 2.07 | 8.54 | 92.58 | 562 | 1020 |
| Ruukki | Ash | 51.0 | 1.90 | 8.82 | 90.93 | 476 | 975 |
| Ruukki | Ash | 51.9 | 2.72 | 7.98 | 92.05 | 520 | 1250 |
| Ruukki | Ash | 46.6 | 2.21 | 8.01 | 82.98 | 4760 | 4100 |
| Ruukki | Ash | 51.8 | 2.70 | 7.89 | 91.56 | 434 | 1240 |
| Lestijärvi | Control | 53.7 | 1.11 | 8.33 | 97.33 | 624 | 493 |
| Lestijärvi | Control | 53.1 | 1.28 | 8.26 | 95.94 | 739 | 571 |
| Lestijärvi | Control | 55.5 | 1.51 | 7.32 | 95.81 | 550 | 704 |
| Lestijärvi | Control | 55.0 | 1.34 | 8.43 | 97.25 | 654 | 515 |
| Lestijärvi | Control | 54.7 | 1.47 | 8.39 | 96.80 | 610 | 604 |
| Lestijärvi | Ash | 53.2 | 1.12 | 9.08 | 98.04 | 586 | 505 |
| Lestijärvi | Ash | 53.0 | 1.26 | 7.71 | 96.28 | 646 | 633 |
| Lestijärvi | Ash | 55.9 | 1.02 | 8.27 | 95.08 | 735 | 622 |
| Lestijärvi | Ash | 55.6 | 1.76 | 6.88 | 94.9 | 665 | 941 |
| Lestijärvi | Ash | 55.8 | 1.61 | 7.26 | 93.70 | 625 | 811 |

Table S2. Descriptive statistics of soil physicochemical properties across sites and treatments.

| **Variable** | **Site** | **Treatment** | **n** | **Mean** | **SD** | **Coefficient of variation** |
| --- | --- | --- | --- | --- | --- | --- |
| Soil carbon (%) | Ala-Akkunus | Ash | 5 | 50.66 | 1.35 | 0.03 |
| Soil carbon (%) | Ala-Akkunus | Control | 5 | 52.04 | 0.72 | 0.01 |
| Soil carbon (%) | Ansasaari | Ash | 5 | 49.44 | 4.12 | 0.08 |
| Soil carbon (%) | Ansasaari | Control | 5 | 53.22 | 1.48 | 0.03 |
| Soil carbon (%) | Lestijärvi | Ash | 5 | 54.70 | 1.47 | 0.03 |
| Soil carbon (%) | Lestijärvi | Control | 5 | 54.40 | 0.98 | 0.02 |
| Soil carbon (%) | Nevajärvi | Ash | 5 | 50.16 | 4.46 | 0.09 |
| Soil carbon (%) | Nevajärvi | Control | 5 | 54.58 | 0.81 | 0.01 |
| Soil carbon (%) | Pelso | Ash | 5 | 52.38 | 1.61 | 0.03 |
| Soil carbon (%) | Pelso | Control | 5 | 53.06 | 0.62 | 0.01 |
| Soil carbon (%) | Ropsajoki | Ash | 5 | 50.60 | 1.13 | 0.02 |
| Soil carbon (%) | Ropsajoki | Control | 5 | 50.68 | 0.65 | 0.01 |
| Soil carbon (%) | Ruukki | Ash | 5 | 50.48 | 2.21 | 0.04 |
| Soil carbon (%) | Ruukki | Control | 5 | 52.44 | 1.01 | 0.02 |
| Soil nitrogen (%) | Ala-Akkunus | Ash | 5 | 1.74 | 0.08 | 0.05 |
| Soil nitrogen (%) | Ala-Akkunus | Control | 5 | 1.61 | 0.18 | 0.11 |
| Soil nitrogen (%) | Ansasaari | Ash | 5 | 1.39 | 0.17 | 0.12 |
| Soil nitrogen (%) | Ansasaari | Control | 5 | 1.50 | 0.12 | 0.08 |
| Soil nitrogen (%) | Lestijärvi | Ash | 5 | 1.35 | 0.32 | 0.24 |
| Soil nitrogen (%) | Lestijärvi | Control | 5 | 1.34 | 0.16 | 0.12 |
| Soil nitrogen (%) | Nevajärvi | Ash | 5 | 1.42 | 0.16 | 0.11 |
| Soil nitrogen (%) | Nevajärvi | Control | 5 | 1.62 | 0.13 | 0.08 |
| Soil nitrogen (%) | Pelso | Ash | 5 | 1.42 | 0.32 | 0.22 |
| Soil nitrogen (%) | Pelso | Control | 5 | 1.17 | 0.12 | 0.10 |
| Soil nitrogen (%) | Ropsajoki | Ash | 5 | 1.94 | 0.18 | 0.09 |
| Soil nitrogen (%) | Ropsajoki | Control | 5 | 1.98 | 0.21 | 0.10 |
| Soil nitrogen (%) | Ruukki | Ash | 5 | 2.32 | 0.37 | 0.16 |
| Soil nitrogen (%) | Ruukki | Control | 5 | 2.12 | 0.45 | 0.21 |
| Soil moisture (%) | Ala-Akkunus | Ash | 5 | 7.64 | 0.22 | 0.03 |
| Soil moisture (%) | Ala-Akkunus | Control | 5 | 6.89 | 0.24 | 0.04 |
| Soil moisture (%) | Ansasaari | Ash | 5 | 6.99 | 0.28 | 0.04 |
| Soil moisture (%) | Ansasaari | Control | 5 | 7.16 | 0.29 | 0.04 |
| Soil moisture (%) | Lestijärvi | Ash | 5 | 7.84 | 0.87 | 0.11 |
| Soil moisture (%) | Lestijärvi | Control | 5 | 8.15 | 0.47 | 0.06 |
| Soil moisture (%) | Nevajärvi | Ash | 5 | 6.78 | 0.47 | 0.07 |
| Soil moisture (%) | Nevajärvi | Control | 5 | 7.52 | 0.58 | 0.08 |
| Soil moisture (%) | Pelso | Ash | 5 | 8.44 | 0.58 | 0.07 |
| Soil moisture (%) | Pelso | Control | 5 | 8.81 | 0.46 | 0.05 |
| Soil moisture (%) | Ropsajoki | Ash | 5 | 6.44 | 0.34 | 0.05 |
| Soil moisture (%) | Ropsajoki | Control | 5 | 6.69 | 0.15 | 0.02 |
| Soil moisture (%) | Ruukki | Ash | 5 | 8.25 | 0.41 | 0.05 |
| Soil moisture (%) | Ruukki | Control | 5 | 8.48 | 0.24 | 0.03 |
| SOM (%) | Ala-Akkunus | Ash | 5 | 92.46 | 1.83 | 0.02 |
| SOM (%) | Ala-Akkunus | Control | 5 | 95.78 | 0.39 | 0.00 |
| SOM (%) | Ansasaari | Ash | 5 | 89.27 | 7.21 | 0.08 |
| SOM (%) | Ansasaari | Control | 5 | 96.46 | 1.19 | 0.01 |
| SOM (%) | Lestijärvi | Ash | 5 | 95.60 | 1.64 | 0.02 |
| SOM (%) | Lestijärvi | Control | 5 | 96.63 | 0.72 | 0.01 |
| SOM (%) | Nevajärvi | Ash | 5 | 91.30 | 8.94 | 0.10 |
| SOM (%) | Nevajärvi | Control | 5 | 97.75 | 0.75 | 0.01 |
| SOM (%) | Pelso | Ash | 5 | 95.77 | 2.46 | 0.03 |
| SOM (%) | Pelso | Control | 5 | 97.87 | 0.68 | 0.01 |
| SOM (%) | Ropsajoki | Ash | 5 | 93.88 | 1.37 | 0.01 |
| SOM (%) | Ropsajoki | Control | 5 | 94.51 | 1.93 | 0.02 |
| SOM (%) | Ruukki | Ash | 5 | 90.02 | 3.98 | 0.04 |
| SOM (%) | Ruukki | Control | 5 | 93.72 | 2.23 | 0.02 |
| Soil P (mg kg^-1^) | Ala-Akkunus | Ash | 5 | 1175.2 | 135.63 | 0.12 |
| Soil P (mg kg^-1^) | Ala-Akkunus | Control | 5 | 797.6 | 48.14 | 0.06 |
| Soil P (mg kg^-1^) | Ansasaari | Ash | 5 | 1237.8 | 724.12 | 0.59 |
| Soil P (mg kg^-1^) | Ansasaari | Control | 5 | 673.8 | 119.07 | 0.18 |
| Soil P (mg kg^-1^) | Lestijarvi | Ash | 5 | 702.4 | 172.46 | 0.25 |
| Soil P (mg kg^-1^) | Lestijarvi | Control | 5 | 577.4 | 83.36 | 0.14 |
| Soil P (mg kg^-1^) | Nevajarvi | Ash | 5 | 1312.8 | 753.25 | 0.57 |
| Soil P (mg kg^-1^) | Nevajarvi | Control | 5 | 799.0 | 101.23 | 0.13 |
| Soil P (mg kg^-1^) | Pelso | Ash | 5 | 806.0 | 166.49 | 0.21 |
| Soil P (mg kg^-1^) | Pelso | Control | 5 | 613.8 | 50.22 | 0.08 |
| Soil P (mg kg^-1^) | Ropsajoki | Ash | 5 | 1316.0 | 221.43 | 0.17 |
| Soil P (mg kg^-1^) | Ropsajoki | Control | 5 | 1027.0 | 84.66 | 0.08 |
| Soil P (mg kg^-1^) | Ruukki | Ash | 5 | 1717.0 | 1337.97 | 0.78 |
| Soil P (mg kg^-1^) | Ruukki | Control | 5 | 1007.6 | 233.63 | 0.23 |
| Soil K (mg kg^-1^) | Ala-Akkunus | Ash | 5 | 1057.80 | 161.21 | 0.15 |
| Soil K (mg kg^-1^) | Ala-Akkunus | Control | 5 | 661.00 | 154.68 | 0.23 |
| Soil K (mg kg^-1^) | Ansasaari | Ash | 5 | 1145.6 | 669.18 | 0.58 |
| Soil K (mg kg^-1^) | Ansasaari | Control | 5 | 422.0 | 133.23 | 0.32 |
| Soil K (mg kg^-1^) | Lestijarvi | Ash | 5 | 651.4 | 55.18 | 0.08 |
| Soil K (mg kg^-1^) | Lestijarvi | Control | 5 | 635.4 | 69.19 | 0.11 |
| Soil K (mg kg^-1^) | Nevajarvi | Ash | 5 | 1206.8 | 1048.85 | 0.87 |
| Soil K (mg kg^-1^) | Nevajarvi | Control | 5 | 628.6 | 111.76 | 0.18 |
| Soil K (mg kg^-1^) | Pelso | Ash | 5 | 666.0 | 327.19 | 0.49 |
| Soil K (mg kg^-1^) | Pelso | Control | 5 | 663.6 | 217.02 | 0.33 |
| Soil K (mg kg^-1^) | Ropsajoki | Ash | 5 | 731.2 | 120.35 | 0.16 |
| Soil K (mg kg^-1^) | Ropsajoki | Control | 5 | 512.2 | 63.42 | 0.12 |
| Soil K (mg kg^-1^) | Ruukki | Ash | 5 | 1350.4 | 1906.62 | 1.41 |
| Soil K (mg kg^-1^) | Ruukki | Control | 5 | 449.2 | 130.44 | 0.29 |
| pH | Ala-Akkunus | Ash | 12 | 4.99 | 0.46 | 0.09 |
| pH | Ala-Akkunus | Control | 12 | 4.27 | 0.19 | 0.04 |
| pH | Ansasaari | Ash | 12 | 4.32 | 0.17 | 0.04 |
| pH | Ansasaari | Control | 12 | 4.13 | 0.08 | 0.02 |
| pH | Lestijarvi | Ash | 12 | 4.08 | 0.11 | 0.03 |
| pH | Lestijarvi | Control | 12 | 3.95 | 0.11 | 0.03 |
| pH | Nevajarvi | Ash | 12 | 4.41 | 0.46 | 0.10 |
| pH | Nevajarvi | Control | 12 | 3.99 | 0.16 | 0.04 |
| pH | Pelso | Ash | 12 | 4.05 | 0.14 | 0.03 |
| pH | Pelso | Control | 12 | 4.01 | 0.23 | 0.06 |
| pH | Ropsajoki | Ash | 12 | 4.52 | 0.20 | 0.04 |
| pH | Ropsajoki | Control | 12 | 4.31 | 0.21 | 0.05 |
| pH | Ruukki | Ash | 12 | 4.24 | 0.19 | 0.05 |
| pH | Ruukki | Control | 12 | 4.20 | 0.18 | 0.04 |

Table S3. Linear mixed‑effects model estimates of temporal development of ash fertilization effects on soil physicochemical variables. Site and treatment nested within site were included as random intercepts.

| **Response variable** | **Estimate^1^** | **SE^1^** | **df^1^** | **T^1^** | **P^1^** | **Estimate^2^** | **SE^2^** | **df^2^** | **T^2^** | **P^2^** |
| --- | --- | --- | --- | --- | --- | --- | --- | --- | --- | --- |
| Soil carbon (%) | 0.093 | 0.031 | 61.34 | 3.01 | 0.004 | -4.12 | 0.93 | 65.59 | -4.45 | < 0.001 |
| Soil nitrogen (%) | 0.0052 | 0.0043 | 5.67 | 1.20 | 0.28 | -0.099 | 0.13 | 5.52 | -0.78 | 0.47 |
| Soil moisture (%) | 0.0029 | 0.013 | 6.20 | 0.23 | 0.83 | -0.26 | 0.38 | 5.93 | -0.70 | 0.51 |
| SOM (%) | 0.12 | 0.051 | 48.86 | 2.38 | 0.022 | -6.64 | 1.56 | 59.50 | -4.26 | < 0.001 |
| Soil P (mg kg^-1^) | -4.21 | 6.95 | 51.72 | -0.61 | 0.55 | 504.69 | 210.80 | 61.03 | 2.39 | 0.020 |
| Soil K (mg kg^-1^) | -9.58 | 7.33 | 67.00 | -1.31 | 0.20 | 653.09 | 237.53 | 67.00 | 2.75 | 0.008 |
| pH | -0.0061 | 0.0057 | 8.16 | -1.07 | 0.31 | 0.41 | 0.17 | 7.34 | 2.38 | 0.047 |

^1^ Fixed effect estimates for the interaction between ash fertilization and years since fertilization, describing how the impact of fertilization develops after the initial impact.

^2^ Fixed effect estimates for ash fertilization, describing the initial effect of fertilization.

Table S4. Variance components for soil physicochemical parameters estimated using mixed-effects models. Variance components represent the percentage of total variance attributable to differences between site, between treatment plots within site, and between individual samples within treatment plot variability. Models included site and treatment nested within site as random effects.

| **Variable** | **Between-site variability (%)** | **Within-site (between plots) variability (%)** | **Within-plot variability (%)** |
| --- | --- | --- | --- |
| Soil carbon (%) | 4.08 | 32.85 | 63.06 |
| Soil nitrogen (%) | 66.17 | 0.46 | 33.37 |
| Soil moisture (%) | 66.64 | 8.52 | 24.83 |
| SOM (%) | 0 | 28.78 | 71.22 |
| Soil P (mg kg^-1^) | 4.93 | 18.36 | 76.71 |
| Soil K (mg kg^-1^) | 0.00 | 2.23 | 97.77 |
| pH | 14.89 | 41.43 | 43.67 |

Table S5. Summary of pairwise PERMANOVA (adonis2) results comparing community composition between two sites. For each site pair, two models were run: (1) a site-only model testing whether sites differ while controlling for treatment; and (2) a treatment only model testing whether treatments differ while controlling for site.

| **Pair** | **Df** | **Site**  **SS** | **Site**  **R^2^** | **Site**  **F** | **Site**  **p** | **Site**  **p-adj** | **Treatment**  **SS** | **Treatment**  **R^2^** | **Treatment**  **F** | **Treatment**  **p** | **Treatment**  **p-adj** |
| --- | --- | --- | --- | --- | --- | --- | --- | --- | --- | --- | --- |
| Ala-Akkunus vs Ansasaari | 1 | 0.455 | 0.226 | 13.408 | 0.001 | 0.001 | 0.145 | 0.072 | 3.568 | 0.001 | 0.003 |
| Ala-Akkunus vs Ropsajoki | 1 | 0.362 | 0.189 | 10.748 | 0.001 | 0.001 | 0.226 | 0.119 | 6.185 | 0.001 | 0.003 |
| Ala-Akkunus vs Ruukki | 1 | 0.517 | 0.271 | 17.108 | 0.001 | 0.001 | 0.152 | 0.080 | 3.993 | 0.001 | 0.003 |
| Ala-Akkunus vs Nevajärvi | 1 | 0.418 | 0.197 | 11.274 | 0.001 | 0.001 | 0.173 | 0.081 | 4.075 | 0.001 | 0.003 |
| Ala-Akkunus vs Lestijärvi | 1 | 0.991 | 0.392 | 29.652 | 0.001 | 0.001 | 0.123 | 0.049 | 2.356 | 0.001 | 0.003 |
| Ala-Akkunus vs Pelso | 1 | 0.821 | 0.331 | 22.774 | 0.001 | 0.001 | 0.094 | 0.038 | 1.818 | 0.002 | 0.004 |
| Ansasaari vs Ropsajoki | 1 | 0.173 | 0.112 | 5.798 | 0.001 | 0.001 | 0.127 | 0.082 | 4.121 | 0.001 | 0.003 |
| Ansasaari vs Ruukki | 1 | 0.175 | 0.126 | 6.641 | 0.001 | 0.001 | 0.061 | 0.044 | 2.109 | 0.005 | 0.009 |
| Ansasaari vs Nevajärvi | 1 | 0.206 | 0.119 | 6.189 | 0.001 | 0.001 | 0.087 | 0.050 | 2.419 | 0.008 | 0.012 |
| Ansasaari vs Lestijärvi | 1 | 0.321 | 0.191 | 10.864 | 0.001 | 0.001 | 0.042 | 0.025 | 1.179 | 0.102 | 0.107 |
| Ansasaari vs Pelso | 1 | 0.215 | 0.127 | 6.669 | 0.001 | 0.001 | 0.050 | 0.029 | 1.384 | 0.064 | 0.075 |
| Ropsajoki vs Ruukki | 1 | 0.160 | 0.118 | 6.136 | 0.001 | 0.001 | 0.122 | 0.090 | 4.538 | 0.001 | 0.003 |
| Ropsajoki vs Nevajärvi | 1 | 0.290 | 0.160 | 8.788 | 0.001 | 0.001 | 0.146 | 0.081 | 4.058 | 0.001 | 0.003 |
| Ropsajoki vs Lestijärvi | 1 | 0.377 | 0.219 | 12.872 | 0.001 | 0.001 | 0.069 | 0.040 | 1.925 | 0.011 | 0.015 |
| Ropsajoki vs Pelso | 1 | 0.360 | 0.197 | 11.276 | 0.001 | 0.001 | 0.112 | 0.061 | 3.012 | 0.002 | 0.004 |
| Ruukki vs Nevajärvi | 1 | 0.272 | 0.167 | 9.207 | 0.001 | 0.001 | 0.072 | 0.044 | 2.131 | 0.006 | 0.010 |
| Ruukki vs Lestijärvi | 1 | 0.358 | 0.231 | 13.843 | 0.001 | 0.001 | 0.064 | 0.041 | 1.977 | 0.003 | 0.006 |
| Ruukki vs Pelso | 1 | 0.437 | 0.250 | 15.351 | 0.001 | 0.001 | 0.058 | 0.033 | 1.572 | 0.013 | 0.017 |
| Nevajärvi vs Lestijärvi | 1 | 0.440 | 0.226 | 13.443 | 0.001 | 0.001 | 0.056 | 0.029 | 1.358 | 0.040 | 0.049 |
| Nevajärvi vs Pelso | 1 | 0.374 | 0.187 | 10.581 | 0.001 | 0.001 | 0.052 | 0.026 | 1.221 | 0.090 | 0.099 |
| Lestijärvi vs Pelso | 1 | 0.187 | 0.114 | 5.906 | 0.001 | 0.001 | 0.043 | 0.026 | 1.246 | 0.138 | 0.138 |

Figure S1. Relative abundances of the 10 most abundant bacterial orders across peatland forest sites and treatments.

Figure S2. Relative abundances of the 10 most abundant bacterial families across peatland forest sites and treatments.
